# Secretion and Detection of Defensive Compounds by the Red Flour Beetle *Tribolium castaneum* Interacting with the Insect Pathogenic Fungus *Beauveria bassiana*

**DOI:** 10.3390/pathogens11050487

**Published:** 2022-04-20

**Authors:** Belén Davyt-Colo, Juan R. Girotti, Andrés González, Nicolás Pedrini

**Affiliations:** 1 Instituto de Investigaciones Bioquímicas de La Plata (INIBIOLP), CCT La Plata Consejo Nacional de Investigaciones Científicas y Técnicas (CONICET)—Universidad Nacional de La Plata (UNLP), calles 60 y 120, La Plata 1900, Argentina; bdavyt@med.unlp.edu.ar; 2 Laboratorio de Ecología Química, Facultad de Química, Universidad de la República. Gral. Flores, Montevideo 2124, Uruguay; agonzal@fq.edu.uy

**Keywords:** chemical ecology, benzoquinones, gene expression, odorant-binding proteins, chemosensory proteins

## Abstract

Entomopathogenic fungi such as *Beauveria bassiana* are extensively used for the control of insect pests worldwide. They infect mostly by adhesion to the insect surface and penetration through the cuticle. However, some insects, such as the red flour beetle *Tribolium castaneum* (Herbst), have evolved resistance by embedding their cuticle with antifungal compounds. Thus, they avoid fungal germination on the cuticle, which result in low susceptibility to entomopathogenic fungi. In adult *T. castaneum*, these antifungals are the well-known defensive compounds methyl-1,4- and ethyl-1,4-benzoquinone. In this study, we added *B. bassiana* conidia on the diet of adult beetles to study the effect of the entomopathogen on the secretion and detection of the beetle volatile blend containing both benzoquinones. The compounds were analyzed by solid phase microextraction coupled to gas chromatography–flame ionization detection, and were detected by electroantennography. In addition, we measured the expression level of four genes encoding for two odorant-binding proteins (OBP), one chemosensory protein (CSP), and one odorant receptor (OR) in both healthy and fungus-treated insects. Significant alterations in the secretion of both benzoquinones, as well as in the perception of methyl-1,4-benzoquinone, were found in fungus-treated insects. *TcOBP7D*, *TcOBP0A* and *TcCSP3A* genes were down-regulated in insects fed conidia for 12 and 48 h, and the latter gene was up-regulated in 72 h samples. *TcOR1* expression was not altered at the feeding times studied. We conclude that fungus-treated insects alter both secretion and perception of benzoquinones, but additional functional and genetic studies are needed to fully understand the effects of fungal infection on the insect chemical ecology.

## 1. Introduction

Insects use volatile organic compounds (VOCs) as chemical cues to recognize and locate vital resources such as food, mate, or enemies [1]. VOCs can act as pheromones when both the sender and receiver of the signal are members of the same species (i.e., intraspecific communication), or as allelochemicals when communication is between organisms of different species (i.e., interspecific communication). Within allelochemicals, there is a group of substances, termed allomones, which provoke a physiological or behavioral response in the receiver that is favorable for the sender [2].

Tenebrionid insects, such as the red flour beetle *Tribolium castaneum* (Herbst) (Coleoptera: Tenebrionidae), use prothoracic and abdominal glands to produce and secrete specific defense compounds with well-known repellent and irritant properties against predators, i.e., acting as allomones [3,4,5]. Methyl-1,4-benzoquinone (MBQ), ethyl-1,4-benzoquinone (EBQ), and 1-pentadecene (C15:1) are the major defensive components of the volatile organic compounds (VOCs) released by adults of this beetle [6]. As both MBQ and EBQ are also perceived by other *T. castaneum* insects, it was proposed that they can act as pheromones, depending on the dose [7,8]. Both benzoquinones can also attract the related beetle *T. confusum* (du Val) in olfactometer assays; however, this attraction begins to decrease when benzoquinone concentration rises [9]. In electroantennographic assays, the response is detected even at those high concentrations, i.e., insects perceive both benzoquinones but are not attracted to them because it can be toxic also for the beetles [9]. In the initial detection of chemical signals, a pool of odorant-binding proteins (OBPs) and chemosensory proteins (CSPs) play an important role in the solubilization and transport of the chemical signal to the odorant receptors (ORs) expressed in the olfactory sensilla on the antennae and palps [10]. A total of 50 OBPs and 20 CSPs have been reported in *T. castaneum*, most of them highly expressed in antennae, heads, mouthparts, legs, and bodies [11]. On the other hand, although 259 OR genes have been described in the beetle genome, only 28 of them are expressed in adults [12]. 

The entomopathogenic fungus *Beauveria bassiana* (Balsamo) Vuillemin (Ascomycota: Hypocreales) is a broad-spectrum insect pathogen able to infect nearly 1000 species [13]. *Beauveria bassiana* mostly starts the infection process by penetration of the insect cuticle, which represents the first encounter and barrier between the host and pathogen. Upon adhesion and recognition of the insect surface, the fungus deploys a combination of biochemical and mechanical tools, including (i) hydrolytic, assimilatory, and detoxifying enzymes, (ii) specialized infection structures, to make its way through the insect integument into the hemocoel, and (iii) secondary metabolites that facilitate hemocoel colonization. Aside from immune, developmental, and behavioral adaptations, some insects produce cuticular compounds to prevent the fungus from attaching and breaching the cuticle [14]. 

Both the volatile blend released by *T. castaneum* and synthetic benzoquinones were able to inhibit germination and growth of *B. bassiana* [15]. It was thus proposed that the beetle’s defensive secretion was the cause for the scarce susceptibility to fungal infections detected in adult beetles [14,15]. Beyond low mortalities observed (between 4 and 20% depending on the strain) [15,16,17,18,19,20], beetles previously dipped in conidial suspensions reduced the secretion of VOCs, but not their glandular production, compared with water-dipped beetles [20], suggesting that the fungus-infected beetle was accumulating the defensive secretion. On the other hand, there is no information regarding either volatile’s perception by *T. castaneum* antennae or expression of genes potentially involved in this process upon infection with entomopathogenic fungi.

In this study, we added *B. bassiana* conidia to the diet of adult beetles and measured VOC secretion and perception in fungus-treated insects, as well as the expression of a suite of genes potentially related to the olfactory perception of chemical signals.

## 2. Results

### 2.1. Volatile Organic Compounds (VOCs) Analysis by Solid Phase Micro Extraction (SPME) Coupled to Gas Chromatography–Flame Ionization Detection (GC-FID)

**Figure 1:**
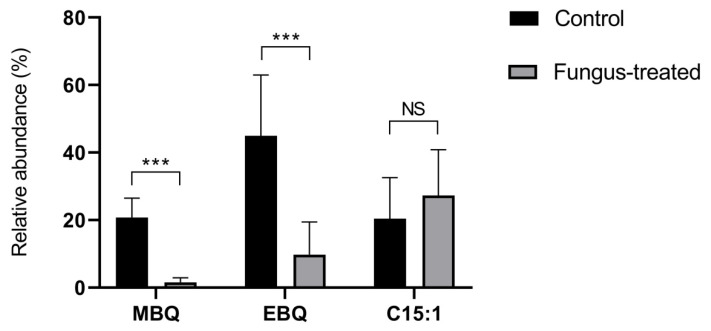
Volatile organic compounds (VOCs) released upon disturbance, measured by solid phase micro extraction coupled to gas chromatography solid phase micro extraction coupled to gas chromatography (SPME-GC). Bars represent mean relative amounts ± SEM. Three adult *T. castaneum* were used in each replicate, and ten replicates were done for each treatment. *** indicates *p* < 0.0005 (Student’s *t*-test), and NS indicates no significant differences.

Both MBQ and EBQ secretions were strongly altered in adult *T. castaneum* fed with *B. bassiana* conidia added to the diet, compared with control insects, while no changes were detected by SPME for 1-pentadecene (C15:1) (Figure 1). The relative amounts of benzoquinones released by *T. castaneum* significantly diminished from 20.7 ± 5.7% to 1.6 ± 1.3% (*p* < 0.0001) (MBQ), and from 45.0 ± 18.0% to 9.8 ± 9.6% (*p* < 0.0005) (EBQ) of the total blend in control and fungus-exposed beetles, respectively. 

### 2.2. VOC Perception by Gas Chromatography–Electroantennographic Detection (GC-EAD)

**Figure 2:**
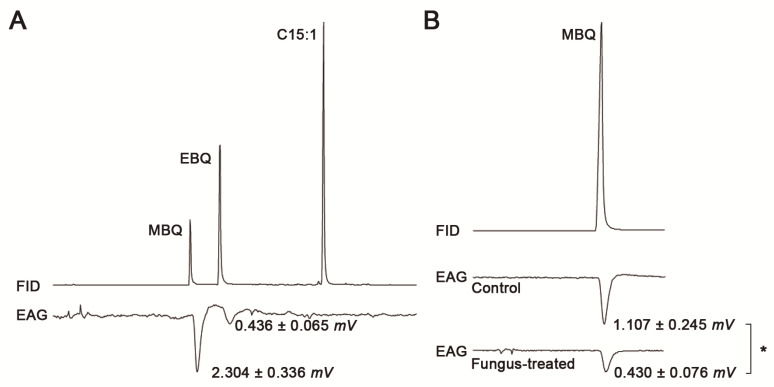
VOC detection by GC-EAD using two antennae of adult beetles. (**A**) Response of antennae from healthy insects (N = 5) to the VOCs released by five healthy insects agitated for 30 s. (**B**) Response of healthy insects (control, N = 3) and fungus-exposed insects (N = 5) to 514 ng/µL MBQ in dichloromethane. In both panels, the upper chromatogram corresponds to the flame ionization detection (FID) signal from the GC, and the lower one corresponds to the electroantennogram signal (EAG) in response to the FID signal. The asterisk (*) indicates *p* < 0.05 (Student’s *t*-test).

Antennae dissected from healthy beetles (control treatment) responded to MBQ (2.30 ± 0.34 mV) and to EBQ (0.44 ± 0.06 mV), but not to C15:1, when they were exposed to the blend release by adult beetles (Figure 2A). As the response to MBQ was higher than the response to EBQ, different concentrations of a commercial MBQ standard were used to establish the appropriate dose to apply to control and fungus-treated beetle antennae. Table 1 shows mean responses of healthy adult antennae (control) to 300, 514, and 1800 ng/µL MBQ. One-way ANOVA showed significant differences among concentrations (*p* = 0.042), Tukey’s post-test evidenced significant differences between 300 and 514 ng/µL (*p* < 0.05), but no significant differences between the latter and 1800 ng/µL. Thus, a solution of 514 ng/µL MBQ was used in the following experiment with fungus-exposed insects. In this case, significant differences (*p* = 0.026, Student’s *t*-test) were found in the perception of MBQ between healthy insects (1.11 ± 0.25 mV) and fungus-exposed insects (0.43 ± 0.08 mV) (Figure 2B).

### 2.3. Gene Expression Analysis 

**Figure 3:**
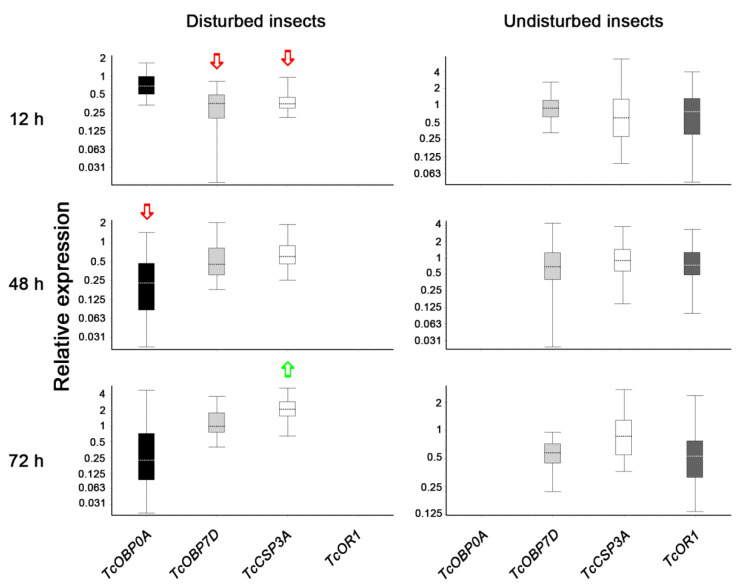
Relative expression analysis of odorant-binding protein (OBP), chemosensory protein (CSP), and odorant receptor (OR) genes in *B. bassiana*-treated *T. castaneum* adults for different time periods compared to healthy insects. The box area encompasses 50% of all observations, the dotted line represents the sample median of three biological replicates, and the vertical bars represent the outer 50% of observations. Green arrow (up-regulation) and red arrow (down-regulation) indicates significant differences (*p* < 0.05).

The relative expression pattern of a suite of genes selected as potentially involved in VOC perception was measured in fungus-treated beetles compared with healthy insects. In both cases, two different treatments were carried out: (i) beetles previously agitated to facilitate VOC secretion (and thus the perception by their peers), and (ii) unagitated insects. Significant changes in the expression of genes encoding for OBP and CSP were found only in fungus-treated, agitated insects (Figure 3). In beetles fed conidia for 12 h, down-regulation was observed for TcOBP7D (*p* = 0.003) and TcCSP3A (*p* = 0.008), whilst TcOBP0A was down-regulated (*p* = 0.025) in beetles fed conidia for 48 h. On the contrary, up-regulation was observed only for TcCSP3A (*p* = 0.023) in insects fed conidia for 72 h. TcOR1, encoding for an odorant receptor coreceptor ortholog of the Drosophila Or83b gene, was found expressed only in non-agitated insects, but—similarly to the rest of the genes measured—no changes in its expression pattern between fungus-treated and healthy insects were found (Figure 3).

## 3. Discussion

*Tribolium castaneum* is a warehouse pest and its mass occurrence is mostly related to human activity. However, the chemical nature of their secretions [21,22,23] and their role in accumulation of individuals with density-dependent effects [24] have been early and extensively explored. Some studies have demonstrated that both benzoquinones (MBQ and EBQ) are differentially produced between individuals by genetic control [25], acting not only as repellents or deterrents, but also as spacing pheromones [26]. Benzoquinones secretion has been proposed as a social dilemma, since due to its properties it first increases the fitness of the colony, but over longer periods of time it becomes detrimental to the group [27]. Thus, in such semi-natural environmental conditions, qualifying a trait as social or not might be a matter of timing [27]. On the other hand, insect exposure to the aggregation pheromone, 4,8-dimethyldecanal, leads to a different pattern of rhythmic locomotive behavior [28]. Therefore, it has been proposed that manipulating the well-adapted “alarm clock” activated by this pheromone may reduce the fitness of insects and thus contribute to pest management [28]. In this last regard, we found that the secretion of both benzoquinones released by *T. castaneum* is altered by the presence of the insect pathogen *B. bassiana* on the insect diet, in concordance with the previous results reported for beetles immersed in conidia suspensions [20]. In other words, *B. bassiana* causes a disturbance on benzoquinones secretion regardless of the infection route, either by cuticle penetration or per os entry. 

We have also verified that *T. castaneum* adults produce electroantennographic signals in response to both natural and synthetic benzoquinones applied to their antennae, in concordance with previous reports [7,8]. The EAD responses recorded here were higher for MBQ than for EBQ; however, the relative response values are not indicative of the real response levels to each compound, since in the experimental design used (GC-EAD), they are applied to the antennae individually after their separation in the chromatographic column. After the first response (in this case to MBQ), the antennal sensibility needs to recover before the next stimulus is offered, to avoid adaptation or a false response to the latter. This recovering time interval depends on the strength of the first stimulus [29]. In nature, beetle antennae find the two compounds simultaneously, thus it can be concluded that both benzoquinones efficiently activate the response system in these insects. We also found that the response to MBQ is dose dependent; making it possible for the beetles to have developed different behavioral responses to different concentrations of benzoquinones, which may reinforce their role as spacing pheromone. In this regard, both MBQ and EBQ released by *T. castaneum* and *T. confusum* attract their co-specifics at low quantities, but high concentrations cause dispersion [9,24]. In turn, we also found that these antennal responses decrease when the entomopathogenic fungus *B. bassiana* is incorporated into the insect’s diet, which may indicate that this spacing adaptive behavior can be altered by fungus infection, by disrupting a functional chemical communication among the insects. Thus, beetles might be attracted when in fact they should be dispersed to avoid adverse situations, e.g., due to poor food availability or inability to detect danger. Such disruptive spacing effect caused by the pathogenic fungus may, in turn, facilitate its dispersion.

Regarding genes potentially involved in odorant perception, we found a different expression pattern related to the time periods in which the insects fed on a conidia-supplemented diet. At short feeding periods, *TcOBP0A*, *TcOBP7D*, and *TcCSP3A* were down-regulated compared with healthy insects, while *TcCSP3A* was up-regulated at the longest feeding period studied. These results should be interpreted with caution, since a detailed tissue-specific transcriptomics study performed in *T. castaneum* has reported that the majority of CSPs and some OBPs are not enriched in antennae or mouthparts, and thus they have a more general role in the transport of hydrophobic molecules [11]. Specifically, these authors reported that CSPs are detected in a wide variety of beetle tissues and are not restricted to the main chemosensory tissues. This could explain the different expression levels of *TcCSP3A*, which might be more related to the presence of hydrophobic metabolites (in this case produced by the fungal infection) than to benzoquinones perception themselves. On the contrary, *TcOBP0A* is reported as enriched in antennae and mouthparts, and *TcOBP7D* showed an atypical, ubiquitous expression but enriched in mouthparts [11]. Thus, the downregulation of *TcOBP0A* in beetles fed with conidia for 48 h suggests that it might play an important role in benzoquinones perception by the antennae, thus contributing to the significant decreases in EAG signals found at 72 h of feeding. *TcOr1* knockdown of by RNAi have demonstrated that this gene plays a structural role in forming the olfactory receptor rather than directly detecting odorants [12], thus it is not surprising that we found this gene expressed only in unagitated insects (where benzoquinones might be either present in very low quantities or absent) and its expression does not change in fungus-treated beetles. The future functional studies planned for OBP encoding genes by using the RNAi approach may shed light on this regard.

In summary, although the entomopathogenic fungus *B. bassiana* generally shows little virulence against *T. castaneum*, it provokes alterations on both secretion and perception of benzoquinones, which might result in changes in the insect’s chemical communication, altering behavior, colony fitness and, ultimately, the entire life cycle of the beetles.

## 4. Materials and Methods

### 4.1. Insects

Adult individuals of *T. castaneum* used in this study came from colonies maintained at the Instituto de Investigaciones Bioquímicas de La Plata (INIBIOLP). Beetles were reared on white wheat flour, with 5% non-fat dried milk, 5% brewer’s yeast and 5% wheat germ, under a 12L:12D photoperiod at 27 ± 2 °C and 70 ± 5% relative humidity. All adults used in the assays were 2-week-old, unsexed insects.

### 4.2. Fungi

The entomopathogenic fungus *B. bassiana* strain ARSEF 2860 was used in this study. The fungus was maintained and routinely grown at 26 °C for 15 days in Potato Dextrose Agar (PDA) medium with 1% ampicillin. Harvested conidia were suspended in 0.01% Tween 80 sterile solution, vortexed, and the concentration was adjusted to 1 × 10^9^ conidia/mL for the downstream insect treatment.

### 4.3. Tribolium Castaneum Treatment with Beauveria bassiana Conidia

To conduct a natural infection process while ensuring close contact between the insects and pathogen, we discarded the traditional in vitro fungal inoculation methods such as insect immersion or spray with a conidia suspension. Instead, the fungus was added as part of the insect diet in a feeding arena. To do that, a mixture of conidia suspension (1 × 10^9^ conidia/mL) and wheat flour was optimized to cover a corn kernel with a thin layer. The mixture was prepared with 5 mL of conidia suspension and 7 mg of wheat flour. Control kernels were similarly prepared, but mixing wheat flour with 0.01% Tween 80 sterile solution instead of the fungal suspension. Kernels were dried at 4 °C for 24 h. One kernel, either fungus-covered or control, was put inside separated Petri dishes and offered to six beetles previously fasted for one day. Insects were observed daily to ensure that they were mounted on and feeding on the kernel. After different time periods detailed below, insects were separated from the feeding arena and used for either volatile assays (72 h) or gene expression analysis (12, 48, and 72 h). 

### 4.4. Volatile Organic Compound (VOC) Analysis by Solid Phase Micro Extraction (SPME) Coupled to Gas Chromatography–Flame Ionization Detection (GC-FID)

Pools of three adults, either healthy or fungus-treated, were gently transferred to a 4-mL glass vial sealed with a Teflon lined rubber septum. After 48 h at 25 °C, the vial was agitated for 30 s to disturb the insects and measure the VOCs secreted, following the protocol described by Villaverde et al. [6]. Volatiles were sampled from the headspace (HS) corresponding to the gaseous phase in contact with the beetle sample by solid phase microextraction (SPME). VOCs were adsorbed by HS-SPME for 15 min at 25 °C by using a 65-µm thickness polydimethylsiloxane/divinylbenzene fiber (Supelco, Bellefonte, PA, USA). Fibers were previously conditioned according to manufacturer instructions. Vials containing kernels were used as a control. VOC analysis was performed using a Hewlett-Packard 6890 gas chromatograph by using a nonpolar DB-5 capillary column (30 m length, 0.32 mm i.d., 0.25-µm thickness) (J&W, Folsom, CA, USA). The injector was operated in the splitless mode at 250 °C. The oven temperature was programmed from 40 °C for 1 min, 20 °C/min to 250 °C, with a holding time of 10 min at the final temperature. The flame ionization detector temperature was set at 280 °C. There were 10 replications of each treatment, with three beetles per replicate. 

### 4.5. Volatile Organic Compound (VOC) Perception by Gas Chromatography–Electroantennographic Detection (GC-EAD) 

The response of *T. castaneum* antennae to VOCs was performed by GC-EAD using an Agilent 6850 gas chromatograph (Agilent Technologies, Santa Clara, CA, USA), employing a non-polar DB-5 capillary column (30 m length, 0.32 mm I.D., 0.25 µm film thickness) (J&W, Folsom, CA, USA) and an outlet splitter system (Agilent Technologies, Santa Clara, CA, USA). An effluent split allowed simultaneous flame ionization detection (FID) and EAD of the separated compounds. Helium was used as carrier gas (1.5 mL/min), and the effluent split ratio was approximately 1:1 to the FID and to the antennae. Run conditions were the same as described above. The transfer line of the effluent conditioner assembly was set at 230 °C (type EC-03, Syntech, Hilversum, The Netherlands). Separated compounds were carried to the antennae through a glass tube by a humidified air stream at 15 mL/s. Both beetle antennae were cut off from the head with a scalpel under a dissecting magnifying glass (Zeiss, Stemi 305, Suzhou, China), then carefully placed in parallel across an antenna electrode holder with an electrode gel (Spectra 360, Parker Laboratory Inc., Orange, NJ, USA), and the holder was connected to the EAG probe (MTP-4, Syntech, Hilversum, The Netherlands). After each run, the electrodes were wiped clean and two new fresh antennae were mounted for the next run. The signal was amplified by intelligent data acquisition controller systems (IDAC-02) and analyzed with GC-EAD software 2010 v.1.2.2 (Syntech, Hilversum, The Netherlands). The IDAC-02 was also connected to the FID such that the antennal responses from the probe and FID outputs could be recorded simultaneously. EAD responses to FID peaks were defined as repeatable deflections of the baseline. 

We performed two experiments, each offering to antennae from control beetles: (i) the total blend release by 5 disturbed beetles and sampled by SPME, containing methyl-1,4- and ethyl-1,4-benzoquinones, and 1-pentadecene [20], and (ii) the commercial standard of methyl-1,4-benzoquinone (Sigma-Aldrich, St. Louis, MO, USA) solution in dichloromethane at three concentrations (300, 514, and 1800 ng/µL). Based on the results of this last experiment (Table 1), the concentration offered to fungus-treated insects was 514 ng/µL. For all experiments, between three and five runs of each condition were conducted.

### 4.6. Identification and Expression Analysis of Potential Perception Genes by Quantitative Real-Time Polymerase Chain Reaction (qRT-PCR)

First, we searched for genes encoding for odorant-binding proteins (OBPs), chemosensory proteins (CSPs), and odorant receptors (ORs) in three transcriptomes of insects fed with conidia-added diet during 12, 48, and 72 h, as detailed in Section 4.3 (the transcriptomics project is available at the European Nucleotide Archive with the accession number PRJEB46827). Then, we selected those genes expressed in the three time points studied, which were identified as OBP (two genes, NCBI accession numbers XM_970591 and XM_008202048), CSP (NCBI accession number NM_001045812), and OR (NCBI accession number XM_008196471). These genes were named according to the new nomenclature for *T. castaneum* OBPs and CSPs proposed by Dippel et al. [11] as *TcOBP7D* (XM_970591), *TcOBP0A* (XM_008202048), *TcCSP3A* (NM_001045812), and the odorant receptor coreceptor was named as *TcOR1* (XM_008196471) according to Engsontia et al. [12]. Finally, their expression pattern was assessed by qRT-PCR. Specific primer sequences used for amplification, including the housekeeping gene (Ribosomal protein 49, *RP49*), are listed in Table 2.

Then, qRT-PCR assays were performed in two insect treatments, each corresponding to insects with and without previous agitation as described in Section 4.4, respectively. For RNA extraction of both insect treatments, pools of ten beetles were used, either control or fungus treated. Total RNA was extracted employing the Tri Reagent^®^ (Molecular Reagent Centre, Cincinnati, OH, USA) technique, according to manufacturer’s instructions. RNA samples were quantified with a Nanodrop spectrophotometer (Thermo, Wilmington, NC, USA), and the integrity was assessed on a 1% (*w*/*v*) agarose gel. One-step real-time polymerase chain reaction (RT-PCR) was carried out with iTaq Universal Sybr Green one step kit (Bio-Rad, Hercules, USA). Amplification was performed on an AriaMx Real-Time PCR (qPCR) Instrument (Agilent Technologies, Santa Clara, CA, USA) employing 100 ng total RNA for each sample. To confirm that only single products were amplified, a temperature-melting step was then performed. Reactions containing primer pairs without a template were included as blank controls. The assay was performed in duplicate for each of the three independent biological replicates. The relative expression ratio, the statistical analysis and the expression plots were done with the REST software (version 2009, Qiagen, Hilden, Germany) [30].

### 4.7. Statistical Analyses

Statistical analyses of the results from Section 4.4 and Section 4.5 were conducted with the GraphPad Prism 8 Software. Student’s *t* test (*p* < 0.05) and analysis of variance (ANOVA) following by Tukey’s test to separate treatment means (*p* < 0.05) were performed. 

## Figures and Tables

**Table 1:** Gas chromatography–electroantennographic detection (GC-EAD) responses of two antennae from healthy beetles to three different concentrations of methyl-1,4-benzoquinone (MBQ).

MBQ (ng/µL)	EAD Response (mV)
300	0.22 ± 0.11 ^a^
514	1.11 ± 0.25 ^b^
1800	0.77 ± 0.32 ^b^

EAD responses represent mean ± SEM (300 ng/µL, N = 5; 514 ng/µL, N = 3; 1800 ng/µL, N = 3). Significant differences in the response to different concentrations are showed by different letters (ANOVA followed by Tukey’s post-test).

**Table 2:** Oligonucleotides used in this study.

Gene (Acronym Used)	Forward (5′-3′)	Reverse (5′-3′)
*TcOBP7D*	TGCTCCTCTTTCTCGCTTTGGC	TTTGGCGTCGTCGGTGAAGTC
*TcOBP0A*	CGTGAAGGCTTCTGCATGCTTG	CGCCGTCTCCCAATTCACTTTC
*TcCSP3A*	CGGGACGTCATTCCAGATGCTC	TGTTGCCAATCGCTGTTGCG
*TcOR1*	GGCGATCAAATACTGGGTGGAG	AACAGCAAATAGCCCAGAACCG
*RP49 * ^1^	TGACCGTTATGGCAAACTCA	TAGCATGTGCTTCGTTTTGG

^1^ Housekeeping gene.

## Data Availability

Not applicable.
